# Association of Mild-to-Moderate Iodine Deficiency With Thyroid Function—A Systematic Review and Meta-analysis

**DOI:** 10.1016/j.advnut.2025.100471

**Published:** 2025-07-08

**Authors:** Tonje Eiane Aarsland, Inger Aakre, Tonje Holte Stea, Sigrun Henjum, Maria Wik Markhus, Tor A Strand, Lisbeth Dahl, Tim IM Korevaar, Kjersti S Bakken, Synnøve Næss Sleire

**Affiliations:** 1 Women’s Clinic at Lillehammer Hospital, Innlandet Hospital Trust, Lillehammer, Norway; 2 Center for International Health, Department of Global Public Health and Primary Care, University of Bergen, Bergen, Norway; 3 Department of Seafood, Nutrition and Environmental State, Institute of Marine Research, Bergen, Norway; 4 Department of Health and Nursing Science, University of Agder, Kristiansand, Norway; 5 Department of Nursing and Health Promotion, Faculty of Health Sciences, Oslo Metropolitan University, Oslo, Norway; 6 Department of Research, Innlandet Hospital Trust, Lillehammer, Norway; 7 Division of Vascular Medicine and Pharmacology, Department of Internal Medicine, Erasmus MC, Rotterdam, Netherlands; 8 Academic Center for Thyroid Diseases, Erasmus University Medical Center, Rotterdam, Netherlands

**Keywords:** iodine, mild-to-moderate iodine deficiency, urinary iodine concentration, thyroid function, thyroid dysfunction, systematic review

## Abstract

The only known function of iodine in the human body is as a component of thyroid hormones. Thus, all consequences of iodine deficiency should be mediated through altered thyroid hormone production. Although it is well established that severe iodine deficiency affects thyroid hormone production, the association between mild-to-moderate iodine deficiency and thyroid function remains unclear. This review aimed to review and summarize observational studies that examine the association between mild-to-moderate iodine deficiency and thyroid hormone function in the general population, including infants, children, adolescents, adults, pregnant and lactating women. Systematic searches of the literature were performed in November 2022 and repeated in February 2024 using the Medline Ovid, Embase Ovid, and Cochrane Central databases. Mild-to-moderate iodine deficiency was defined as a median urinary iodine concentration (UIC) of 20–100 μg/L in children, adolescents, and general adults, and 50–150 μg/L in pregnant women. Thyroid function outcomes included thyroid-stimulating hormone (TSH), free thyroxine (fT3), and free triiodothyronine (fT4) and clinical thyroid dysfunction entities. A total of 72 studies were included: 59 cross-sectional, 12 repeated cross-sectional (longitudinal), and 1 cohort study. Populations studied included infants, children, and adolescents (*n* = 7); women of reproductive age, including lactating women (*n* = 5); general adults (*n* = 20); and pregnant women (*n* = 43). For all population groups, most studies found no clear association between iodine status and thyroid function. Meta-analyses for 8 studies in pregnant women showed no difference in TSH, fT4, or fT3 for those with mild-to-moderate iodine deficiency compared with adequate status [mean difference (95% CI): TSH, 0.03 (−0.05, 0.12) mIU/L; fT4, −0.20 (−0.94, 0.53) pmol/L; fT3, 0.05 (−0.14, 0.03) pmol/L]. In conclusion, no clear association between mild-to-moderate iodine deficiency and thyroid function in the different population groups was found.

This trial was registered at www.crd.york.ac.uk/PROSPERO as CRD42022360447.

**Statement of significance**

This is the first systematic review to provide a comprehensive overview of observational studies investigating the association between mild-to-moderate iodine deficiency and thyroid function in the general population, including infants, children, adolescents, adults, pregnant and lactating women.

## Introduction

Iodine is an essential micronutrient required to produce thyroid hormones, including triiodothyronine (T3) and thyroxine (T4), which are vital components for normal growth and brain development [1]. Therefore, the health consequences of inadequate iodine nutrition are likely mediated through inadequate thyroid hormone production throughout life [2]. Specifically, severe iodine deficiency during fetal life and early childhood, resulting in hypothyroidism, is a well-known cause of impaired neurodevelopment [2]. However, the evidence for whether mild-to-moderate iodine deficiency is associated with altered thyroid hormone production is less clear, and the mechanisms for the association between mild-to-moderate iodine deficiency and negative health outcomes (e.g., neurodevelopment) are not clearly understood due to the missing link with thyroid hormone production [3].

Although severe iodine deficiency has been almost completely eradicated globally because of salt iodization programs and dietary iodine supplementation [4], mild-to-moderate iodine deficiency is still common in many countries, particularly among pregnant women, women of reproductive age, and infants [5]. Previous systematic reviews and meta-analyses have reported lack of good-quality evidence, especially well-designed randomized controlled trials (RCTs), to support current recommendations for iodine supplementation in pregnancy in areas of mild-to-moderate deficiency [6,7]. However, several observational studies have linked mild-to-moderate iodine deficiency in pregnancy to impaired child neurodevelopment [[6], [7], [8], [9], [10], [11]]. If mild-to-moderate iodine deficiency in pregnancy leads to impaired child neurodevelopment, it would be expected that this was mediated via changes in thyroid hormones. Unfortunately, most observational studies reporting an association between mild-to-moderate iodine deficiency during pregnancy and impaired child neurodevelopment do not report data on thyroid function [[9], [10], [11], [12], [13]]. Furthermore, results from the published studies in this field are inconsistent, as some have found an association between mild-to-moderate iodine deficiency and thyroid function [[14], [15], [16]], whereas others have not [[17], [18], [19]]. Moreover, it is still uncertain at which level of iodine status the production of thyroid hormones is affected [3], and the cutoffs to define mild-to-moderate iodine deficiency are not consistently applied.

Previous systematic reviews and meta-analyses have examined the impact of iodine supplementation on thyroid function in populations with mild-to-moderate iodine deficiency. These systematic reviews have consistently found insufficient evidence to conclude whether iodine supplementation for mild-to-moderate iodine deficiency affects thyroid hormone function [6,7,[20], [21], [22]]. In order to fill gaps in the literature, as previous systematic reviews focused only on interventions with iodine supplementation, this review aimed to investigate and review observational studies reporting on the association between iodine status and thyroid function in populations with mild-to-moderate iodine deficiency. In this review, we summarize the evidence for this association in the general population, including infants, children, adolescents, adults, pregnant and lactating women, and older adults.

## Methods

This systematic review was conducted in accordance with the guidelines from the PRISMA 2020 guidelines. The systematic review is registered in the PROSPERO (CRD42022360447), and a prespecified study protocol was published online: https://www.crd.york.ac.uk/prospero/display_record.php?ID=CRD42022360447. Deviation from the protocol is given in Supplemental Table 1.

### Search strategy and eligibility criteria

Literature searches were performed in Medline Ovid, Embase Ovid, and Cochrane Central databases on 2 November, 2022, with no restrictions on publication dates. The searches were repeated on 12 February, 2024, to capture relevant publications since the initial searches. The search strategy is available in the Supplemental Table 2. The Population, Exposure, Comparator, Outcome and Study design diagram used to develop the search strategy and eligibility criteria is shown in Table 1 [23,24]. The literature search strategy was developed together with a librarian at the University of Bergen.

**TABLE 1:** Eligibility criteria for population/participants, exposure, comparison, outcome and study design (PECOS).

Population/participants	General population groups, including infants, children and adolescents, women of reproductive age, adults, pregnant, and lactating women
Exposure	Participants with mild-to-moderate iodine deficiency^1^ measured by iodine nutrition status: - UIC (μg/L) or UIC:Cr (μg/g) - Dietary iodine intake (μg/d) converted to UIC^2^
Comparison	Participants with adequate iodine status^3^
Outcome	Thyroid function outcomes including the following: - TSH - (f)T3 - (f)T4 - Thyroid dysfunction (prevalence of overt/subclinical hypothyroidism, overt/subclinical hyperthyroidism, hypothyroxinaemia, and hyperthyroxinemia)
Study design	Observational studies - Cross-sectional studies - Prospective and retrospective cohort studies - Case–control studies

Abbreviations: Cr, creatinine; fT3, free triiodothyronine; fT4, free thyroxine; T3, triiodothyronine; T4, thyroxine; TSH, thyroid-stimulating hormone; UIC, urinary iodine concentration.

1 Median UIC in the whole study population corresponding to mild-to-moderate iodine deficiency or a distinct group with mild-to-moderate iodine deficiency presented in the study (defined as UIC of 20–100 μg/L in infants, children, adolescents, and adults and 50–150 μg/L in pregnant women) [23,24].

2 Did not apply to any of the included studies.

3 Median UIC of 100–199 μg/L in school-age children and general adults, ≥100 μg/L in lactating women and children <2 y of age, and 150–249 μg/L in pregnant women [23].

Inclusion criteria for articles were that they investigated iodine nutrition [measured as urinary iodine concentration (UIC) or UIC:Cr, or presented as iodine intake converted to UIC] in relation to 1 or more outcomes of thyroid function [free triiodothyronine (fT3), free thyroxine (fT4), T3, T4, thyroid-stimulating hormone (TSH), or prevalence of thyroid dysfunction], included participants with mild-to-moderate iodine deficiency, and compared these with adequate iodine status. Studies were included if either the median UIC in the whole study population corresponded to mild-to-moderate iodine deficiency or if a distinct group within the study population were defined as mildly to moderately iodine deficient. Iodine status was defined according to UIC or UIC:Cr. Adequate iodine status was defined according to the WHO epidemiological criteria for assessing iodine nutrition as median UIC of 100–199 μg/L in school-age children and general adults, ≥100 μg/L in lactating women and children <2 years of age, and 150–249 μg/L in pregnant women [23]. For children, adolescents, and general adults, a median UIC of 20–100 μg/L was defined as mild-to-moderate iodine deficiency according to the WHO epidemiological criteria for assessing iodine nutrition [23]. For pregnant women, there are currently no established categories for the degree of iodine deficiency. Thus, we defined mild-to-moderate iodine deficiency as a median UIC of 50–150 μg/L, as is typically done [24]. Although the focus in this review was on mild-to-moderate iodine deficiency and thyroid function, many studies reported thyroid function outcomes without separating between those with severe iodine deficiency and those with milder deficiency. Thus, for the systematic review, we also included publications that gave thyroid function outcomes for wider UIC groups (e.g., UIC <100 μg/L in children, adolescents, and general adults and <150 μg/L in pregnant women), given that the median UIC within that group corresponded to mild-to-moderate iodine deficiency according to the definitions given earlier.

Exclusion criteria were studies with an experimental design where iodine was given as an intervention (e.g., administration of dietary iodine supplementation, iodized salt, or iodine-rich foods), studies where participants used iodine-containing drugs, medications, or contrast medium, radioactive iodine, or povidone–iodine for disinfection, studies focusing on specific patient or population groups (e.g., patients with cancer or kidney disease, patients receiving parenteral nutrition, or vegans), and case reports, narrative reviews/commentary articles, and other systematic reviews or meta-analyses. Furthermore, studies in languages other than English were excluded.

### Study selection and data extraction

Screening of titles and abstracts were done by 2 independent reviewers (TEA and SNS) using Rayyan (https://www.rayyan.ai). Articles that appeared to fulfill the inclusion criteria upon title and abstract screening were subsequently assessed in full text for their suitability for data extraction. Disagreements between the reviewers were resolved through discussion or by discussion with 3 other members of the study team (IA, KSB, and TS). Relevant data from the included articles were extracted into a predefined table, including reference details (first author, year, and country), study details (aim, study design, and setting), population details (population group, age, sex, and sample size), inclusion and exclusion criteria, iodine status information [median (IQR) and/or mean (SD/SE), urine sampling procedure, and analytical method of iodine status], thyroid function tests measured, association between UIC or UIC:Cr and thyroid hormone function, confounding variables adjusted for, and study sponsor and financial statement.

### Quality assessment of eligible studies

Risk of bias was assessed independently by 2 reviewers (TEA and SNS) using the Newcastle–Ottawa Scale (NOS) adapted for cross-sectional studies [25]. This tool was chosen for quality assessment since most of the included studies were cross-sectional or repeated cross-sectional studies. Each article was given 0–10 stars based on elements from 3 dimensions of bias (selection, comparability, and outcome assessment). Based on the total number of stars given of 10 possible, risk of bias was assessed as high (0–4), moderate (5–6), or low (7–10). Any disagreement between the reviewers was resolved through discussion. No studies were excluded based on risk of bias assessment.

### Synthesis methods and meta-analyses

A qualitative synthesis of all included articles were performed by describing the main characteristics and the overall results of the study. To combine results, the articles were divided into 4 groups according to population group: *1*) infants, children, and adolescents; *2*) women of reproductive age (including lactating women); *3*) general adults; and *4*) pregnant women.

For the meta-analysis, we included only articles that examined thyroid function outcomes exclusively for groups with mild-to-moderate iodine deficiency compared with those with adequate status within the same study population (cutoffs given in Table 1). Further, sample mean and SD for the thyroid function outcomes were extracted. If not given in the article, mean and SD were estimated using the median, IQR, and sample size [26]. Moreover, measurements of the outcomes were converted to consistent units (milli-international units per liter for TSH and picomoles per liter for fT3 and fT4) using known conversion factors. Random-effect meta-analyses were conducted in STATA/SE 16.1 (StataCorp) using the restricted maximum likelihood technique. Further, the mean difference in each qualified study was calculated by subtracting the mean concentration of the thyroid function outcome in the iodine-adequate group from the mean concentration in the mild-to-moderate group. Thus, a mean difference with a negative sign indicates a higher concentration in the adequate group, whereas a mean difference with a positive sign indicates a higher concentration in the mild-to-moderate group.

The proportion of heterogeneity in the meta-analyses was assessed statistically by using the *I*^2^ index (range: 0%–100%) [27]. *I*^2^ values of 25%, 50%, and 75% were considered low, moderate, and high, respectively [27]. Because of the limited number of studies included in the meta-analyses, it was not possible to perform any further subgroup analyses to explain heterogeneity between the results. Publication bias of the studies included in meta-analyses was assessed by visual inspection of funnel plots conducted in STATA/SE 16.1 (StataCorp). We did not perform statistical test for funnel plots asymmetry due to the low number of studies included [28].

### Grading of evidence

Grading of evidence was performed using the criteria from the World Cancer Research Fund (WCRF) [29] for each of the thyroid function outcomes [TSH, (f)T3, (f)T4, and thyroid dysfunction] in the specific population groups. The categories from the WCRF for grading evidence were convincing, probable; limited—suggestive; limited—no conclusion; or substantial effect on risk unlikely [29].

## Results

### Study selection and search results

**FIGURE 1:**
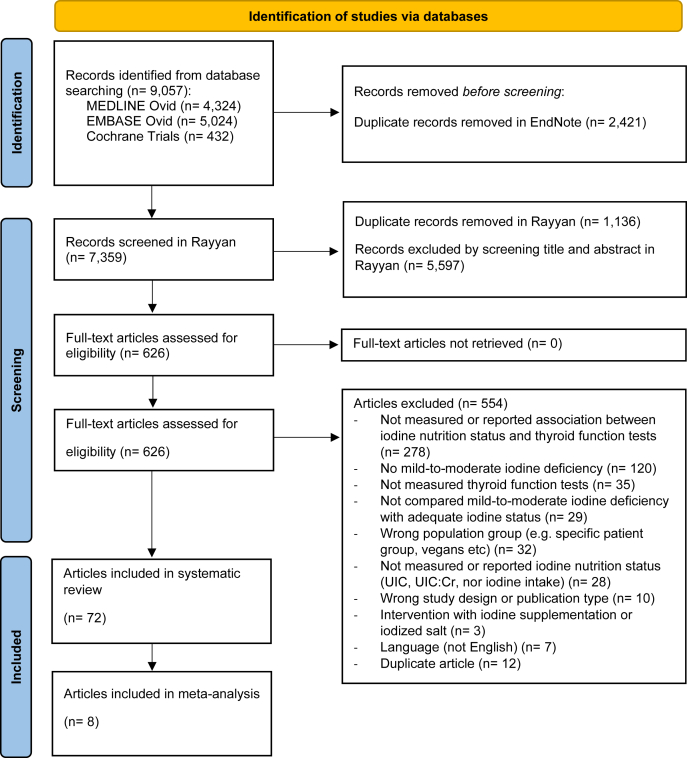
PRISMA diagram showing the flow of information through the different phases of the systematic review. UIC, urinary iodine concentration; UIC:Cr, urinary iodine:creatinine ratio.

The literature searches yielded 9057 records from the 3 databases, whereas 7359 records were screened for title and abstract after removal of duplicates (Figure 1). A total of 626 articles were assessed in full text, where 554 were excluded (Figure 1; list of excluded studies with reason for exclusion is given in Supplemental Table 3). Finally, 72 articles were included in the systematic review for qualitative synthesis, and of these, 8 were included in quantitative analyses (meta-analyses).

### Characteristics of the included studies

The included 72 papers were published between 2007 and 2023. Of these, 7 studies included infants, children, and/or adolescents (Table 2) [[30], [31], [32], [33], [34], [35], [36]], 5 studies included women of reproductive age (including lactating women) (Table 3) [[36], [37], [38], [39], [40]], 20 studies included general adults (Table 4) [36,[41], [42], [43], [44], [45], [46], [47], [48], [49], [50], [51], [52], [53], [54], [55], [56], [57], [58], [59]], and 43 studies included pregnant women (Table 5) [[14], [15], [16], [17], [18],^36^,[60], [61], [62], [63][14–18,36,60–63,[64], [65], [66], [67], [68], [69], [70], [71], [72], [73], [74], [75], [76], [77], [78], [79], [80], [81], [82], [83], [84], [85], [86], [87], [88], [89], [90], [91], [92], [93], [94], [95], [96]. One study [36] reported results separately for all these population groups (TABLE 2, TABLE 3, TABLE 4, TABLE 5). Further, 58 studies had a cross-sectional design, 13 studies had a longitudinal design with repeated cross-sectional measurements, and 1 study was a cohort study.

**TABLE 2:** Characteristics and summary of findings of included studies for the population group infants, children and adolescents (*n* = 7).

Reference; country	Study design and population	Iodine status (UIC), median UIC (IQR) (μg/L)^1^	Association between iodine status and thyroid function outcomes^2^				Risk of bias^3^	Overall results^4^
			TSH	fT3/T3	fT4/T4	Thyroid dysfunction		
Cui et al., 2020 [30]; China	Cross-sectional; *n* = 498; 7–12 y	Not given	No effect	NA	NA	NA	High	No difference in TSH between UIC groups of <100 and 100–300 μg/L.
Jukic et al., 2015 [31]; Croatia	Cross-sectional; *n* = 159; 6–12 y	205 (range 1–505)	No effect	NA	NA	NA	High	No difference in TSH or T4 between UIC groups of <50, 50–99, and 100–199 μg/L.
Meng et al., 2013 [36]; China	Cross-sectional; *n* = 627; mean (SD) age: 10 (2) y	271	↑	NA	NA	NA	High	Higher TSH in UIC groups of <100 vs. 100–199 μg/L.
Næss et al., 2023 [32]; Norway	Longitudinal study with repeated cross-sectional data; 3 mo: *n* = 47; 6 mo: *n* = 53	3 mo: 82 (54–140) 6 mo: 110 (78–190)	No effect	No effect/NA	No effect/NA	NA	Low	No associations between UIC and TSH, fT3, or fT4 in linear regression analyses.
Skeaff et al., 2012 [33]; New Zealand	Cross-sectional; *n* = 1153; 5–14 y	68 (50–95)	No effect	↑/NA	No effect/NA	NA	Low	Higher fT3 in UIC group <50 vs. ≥50 μg/L and in UIC group <100 vs. ≥100 μg/L; no difference in TSH or fT4 between the UIC groups. Thyroid function tests were within normal reference ranges.
Wallborn et al., 2021 [34]; Germany	Cross-sectional; *n* = 1802; 0–17 y	87 for boys 80 for girls	No effect	↑/NA	No effect/NA	NA	High	Negative correlation between UIC with fT3, though a weak correlation; no correlation with TSH or fT4.
Zou et al., 2014 [35]; China	Cross-sectional; *n* = 131; 8–10 y	183 (range, 1345)	No effect	No effect/no effect	No effect/no effect	NA	High	No difference in fT3, T3, fT4, T4, or TSH between UIC groups of <100 and 100–300 μg/L.

fT3, free triiodothyronine; fT4, free thyroxine; NA, not applicable; T3, triiodothyronine; T4, thyroxine; TSH, thyroid-stimulating hormone; UIC, urinary iodine concentration.

1 Median (IQR) UIC in the whole study population. If median and/or IQR value is not given, this is specified.

2 The association between iodine status and thyroid function outcomes. The arrows indicate the association between iodine status and thyroid function outcomes, where ↑ indicates higher concentration/prevalence in the iodine-deficient group than that in the adequate-status group, and ↓ indicates lower concentration/prevalence in the iodine-deficient group than that in the adequate-status group.

3 Risk of bias assessed with the Newcastle–Ottawa Scale and scored in the categories high, medium, and low risk of bias. Full risk of bias assessment is given in Supplemental Table 5.

**TABLE 3:** Characteristics and summary of findings of included studies for the population group women of reproductive age (including lactating women) (*n* = 5).

Reference; country	Study design and population	Iodine status (UIC), median UIC (IQR) (μg/L)^1^	Association between iodine status and thyroid function outcomes^2^				Risk of bias^3^	Overall results^4^
			TSH	fT3/T3	fT4/T4	Thyroid dysfunction		
Hollowell and Haddow, 2020 [37]; USA	Cross-sectional; *n* = 5057; 15–44 y	127 (SD: 4)	No effect	NA	NA/No effect	NA	Moderate	No difference in TSH or T4 between UIC groups <100 vs. 100–299 μg/L.
Kumorowulan et al., 2020 [38]; Indonesia	Cross-sectional; *n* = 748; 15–45 y	174 (range: 1–1690)	No effect	NA	↓/NA	No effect	High	Lower fT4 in UIC group <100 vs. 100–299 μg/L. No clear difference in TSH or prevalence of hyper- and hypothyroidism between UIC groups.
Meng et al., 2013 [36]; China	Cross-sectional; *n* = 332; mean (SD) age: 26 (4) y; lactating women	194	No effect	NA	NA	NA	High	No difference in TSH between UIC groups <100 vs. 100–199.9 μg/L.
Pal et al., 2018 [39]; India	Cross-sectional; *n* = 128; 17–31 y; lactating women ≤3 mo postpartum	185 (range: 17–305)	No effect	NA	No effect/NA	NA	High	No difference in TSH or fT4 between UIC group of <100 vs. 100–199 μg/L.
Soldin et al., 2005 [40]; United States	Cross-sectional; *n* = 2,750; 15–44 y	138 (range: 5–1460)	↓	NA	NA/no effect	NA	High	TSH was lower in UIC:Cr groups of 0–50 and 50.01–100 μg/g vs. 100.01–200 and ≥200 μg/g. This difference was not observed for UIC alone.

Cr, creatinine; fT3, free triiodothyronine; fT4, free thyroxine; NA, not applicable; T3, triiodothyronine; T4, thyroxine; TSH, thyroid-stimulating hormone; UIC, urinary iodine concentration.

1 Median (IQR) UIC in the whole study population. If median and/or IQR value is not given, this is specified.

2 The association between iodine status and thyroid function outcomes. The arrows indicate the association between iodine status and thyroid function outcomes, where ↑ indicates higher concentration/prevalence in the iodine-deficient group than that in the adequate-status group, and ↓ indicates lower concentration/prevalence in the iodine-deficient group than that in the adequate-status group.

3 Risk of bias assessed with the Newcastle–Ottawa Scale and scored in the categories high, medium, and low risk of bias. Full risk of bias assessment is given in Supplemental Table 5.

**TABLE 4:** Characteristics and summary of findings of included studies for the population group general adults (*n* = 20).

Reference; country	Study design and population	Iodine status (UIC), median UIC (IQR) (μg/L)^1^	Association between iodine status and thyroid function outcomes^2^				Risk of bias^3^	Overall results^4^
			TSH	fT3/T3	fT4/T4	Thyroid dysfunction		
Andersen et al., 2009 [41]; Denmark	Cross-sectional; *n* = 430; 75–80 y	Mild iodine deficiency (Randers): 55 (36–98) Adequate status (Skagen): 160 (126–228)	↓	No effect/NA	↓/NA	↑	Moderate	Lower TSH and fT4, and higher prevalence of hyperthyroidism in the population with median UIC of 55 μg/L vs. the population with median UIC of 160 μg/L.
Chen et al., 2019 [42]; China	Cross-sectional; *n* = 14,230; >15 y	205 (95% CI: 66–538)	↑	No effect/NA	↑/NA	NA	Moderate	Higher TSH and fT4 in UIC group of <100 vs. 100–199 and 200–299 μg/L.
Chen et al., 2022 [43]; China	Cross-sectional; *n* = 2877; 18–75 y	Shanghai: 200 (143–292) Wuqiang: 213 (150–291)	No effect	NA/no effect	NA/no effect	NA	Low	No difference in TSH, T3, or T4 between UIC groups of <100 and 100–199 μg/L.
Du et al., 2014 [44]; China	Cross-sectional; *n* = 2147; mean (SD) age—sufficient: 58 (12) y; deficient: 52 (15) y;	Sufficient: 229 (134–323) Deficient: 62 (36–99)	NA	NA	NA	↑	High	Lower prevalence of subclinical hypothyroidism, but higher prevalence of subclinical and overt hyperthyroidism in the UIC group of <100 vs. 100–300 μg/L. No difference in the prevalence of overt hypothyroidism.
Fan et al., 2023 [45]; China	Cross-sectional; *n* = 2628; >18 y	218 (148–313)	NA	NA	NA	↑	High	Higher prevalence of clinical hypothyroidism in the UIC group of <100 vs. ≥100 μg/L. No difference in the prevalence of other thyroid dysfunction outcomes.
Guo et al., 2016 [46]; China	Cross-sectional; *n* = 1591; 18–84 y	131	NA	NA	NA	↑	High	Higher prevalence of overt hyperthyroidism and hypothyroidism in thr UIC group of <100 vs. 100–300 μg/L. No difference in the prevalence of subclinical hyperthyroidism and hypothyroidism.
Jain, 2013 [47]; United States	Cross-sectional; *n* = 1540; ≥12 y	Not given	↑	No effect/↑	No effect/↑	NA	High	Higher TSH, TT3, and TT4 in the UIC group of <100 vs ≥100 μg/L. No difference in fT3.
Kim et al., 2017 [49]; South Korea	Cross-sectional; *n* = 4181; ≥10 y, median age: 41 y	310 (160–750)	NA	NA	NA	No effect	Moderate	No difference in the prevalence of thyroid dysfunction between UIC groups of <100 and 100–300 μg/L.
Kim et al., 2019 [48]; South Korea	Cross-sectional; *n* = 6564; ≥6 y (86% of them, >19 y)	294 (157–684)	↓	NA	No effect/NA	NA	Low	Lower TSH in the UIC group of <100 vs. 100–199 and 200–299 μg/L. No difference in fT4.
Li et al., 2020 [50]; China	Cross-sectional; *n* = 78,470; ≥18 y	178 (118–264)	NA	NA	NA	↑	Low	Higher prevalence of overt hyperthyroidism and subclinical hypothyroidism in the UIC group of <100 vs. 100–199 μg/L. No difference in the prevalence of overt hypothyroidism and subclinical hyperthyroidism.
Meng et al., 2013 [36]; China	Cross-sectional; *n* = 699; mean (SD) age adults: 35 (7) y	260	↑	NA	NA	NA	High	Higher TSH in the UIC group of <100 vs. 100–199 μg/L.
Ning et al., 2020 [51]; China	Cross-sectional; *n* = 2160; ≥18 y	154 (99–229)	NA	NA	NA	No effect	High	No difference in the prevalence of overt and subclinical hyperthyroidism, and overt and subclinical hypothyroidism between UIC groups of <100 and 100–199 μg/L.
Ren et al., 2021 [52]; China	Cross-sectional; *n* = 2020; 18–60 y	245 (149–389)	↑	No effect/NA	No effect/NA	NA	High	Higher TSH in the UIC group of <100 vs. 100-299 μg/L. No difference in fT3 or fT4.
Roef et al., 2013 [53]; Belgium	Cross-sectional; *n* = 941; 25–45 y	74 (54–99)	No effect	No effect	↓/No effect	NA	Moderate	UIC was not associated with TSH, (f)T3, or (f)T4 in linear mixed models. UIC:Cr was negatively associated with fT4.
Vargas-Uricoechea et al., 2022 [54]; Colombia	Cross-sectional; *n* = 412; ≥18 y	154 (220)	No effect	NA	NA	NA	High	No difference in TSH between UIC groups of <100 and 100–199 μg/L.
Wang et al., 2019 [55]; China	Cross-sectional; *n* = 819; ≥18 y	134 (87–201)	No effect	No effect/NA	No effect/NA	NA	High	No difference in TSH, fT3, or fT4 between UIC groups of <100 and 100–299 μg/L.
Xu et al., 2022 [56]; China	Cross-sectional; *n* = 611; mean (SD) age: 43 (16) y	156 (95–211)	NA	NA	NA	↑	Moderate	Higher prevalence of overt and subclinical hypothyroidism in UIC group <100 vs. 100–300 μg/L in women. No differences in men.
Yan et al., 2023 [57]; China	Cross-sectional; *n* = 2636; ≥18 y	176	NA	NA	NA	No effect	Moderate	No difference in risk of thyroid dysfunction between UIC group <100 μg/L and 100–199 μg/L.
Zhao et al., 2020 [59]; China	Cross-sectional; *n* = 78,470; ≥18 y	Not given	↓	NA	NA	No effect	Moderate	Lower TSH in the UIC group of <50 vs. 50–99 and 100–199 μg/L. No clear differences in the prevalence of thyroid dysfunction between UIC groups.
Zhao et al., 2021 [58]; China	Cross-sectional; *n* = 2691; ≥18 y	165 (112–237)	No effect	NA	NA	NA	High	No difference in TSH between UIC groups of <100 and 100–199 μg/L.

Cr, creatinine; fT3, free triiodothyronine; fT4, free thyroxine; NA, not applicable; T3, triiodothyronine; T4, thyroxine; TSH, thyroid-stimulating hormone; UIC, urinary iodine concentration.

1 Median (IQR) UIC in the whole study population. If median and/or IQR value is not given, this is specified.

2 The association between iodine status and thyroid function outcomes. The arrows indicate the association between iodine status and thyroid function outcomes, where ↑ indicates higher concentration/prevalence in the iodine-deficient group than that in the adequate-status group, and ↓ indicates lower concentration/prevalence in the iodine-deficient group than that in the adequate-status group.

3 Risk of bias assessed with the Newcastle–Ottawa Scale and scored in the categories high, medium, and low risk of bias. Full risk of bias assessment is given in Supplemental Table 5.

**TABLE 5:** Characteristics and summary of findings of included studies for the population group: pregnant women (*n* = 43).

Reference; country	Study design and population	Iodine status (UIC), median UIC (IQR) (μg/L)^1^	Association between iodine status and thyroid function outcomes^2^				Risk of bias^3^	Overall results^4^
			TSH	fT3/T3	fT4/T4	Thyroid dysfunction		
Abel et al., 2018 [15]; Norway	Cross-sectional; second trimester: *n* = 2910	68 (35–116)	No effect	↑/NA	↑/NA	NA	Low	UIC was negatively associated with fT3 and fT4 in adjusted cubic splines, but not with TSH, although the effect sizes were small.
Aguyano et al., 2013 [60]; Spain	Longitudinal study with repeated cross-sectional data; first trimester: *n* = 2104; second trimester: *n* = 1322	First trimester: 89 (range: 16–875) second trimester: 140 (range: 21–880)	↓	↑/NA	No effect/NA	NA	High	A weak negative correlation was seen between UIC and fT3 in first trimester, and a weak positive association was seen between UIC and TSH in second trimester.
Alvarez-Pedrerol et al., 2009 [61]; Spain	Cross-sectional; first trimester: *n* = 251	95 (56–154)	No effect	NA	NA/no effect	NA	Moderate	No linear relationship between different UIC groups (<50, 50–99, 100–149, and 150–249 μg/L) and TSH or fT4.
Amouzegar et al., 2013 [62]; Iran	Longitudinal study with repeated cross-sectional data; first trimester: *n* = 203; second trimester: *n* = 201; third trimester: *n* = 197	First trimester: 218 (150–276) Second trimester: 160 (106–260) Third trimester: 145 (88–225)	No effect	NA/no effect	NA/no effect	NA	High	No correlation was found between UIC and TSH, T3, or T4.
Bath et al., 2017 [63]; United Kingdom	Longitudinal study with repeated cross-sectional data; first trimester: *n* = 191; second trimester: *n* = 186; third trimester: *n* = 177	First trimester: 39 (23–84) Second trimester: 55 (32–103) Third trimester: 73 (45–127)	No effect	NA	NA	NA	Moderate	No difference in TSH between UIC:Cr groups <100, 100–149, and 150–249 μg/g.
Berg et al., 2017 [16]; Norway	Longitudinal study with repeated cross-sectional data; second trimester, 3 d postpartum and 6 wk postpartum: *n* = 197	UIC:Cr (μg/g): second trimester: 84; 3 d postpartum: 39; 6 wk postpartum: 41	NA	↑/↑	↑/NA	NA	Low	Compared with UIC:Cr group >150 μg/g, the groups with UIC:Cr 100–149 and <100 μg/g were associated with higher T3, fT3, and fT4, although the concentrations varied within normal reference ranges.
Blumenthal et al., 2012 [64]; Australia	Cross-sectional; first trimester: *n* = 367	81 (41–167)	No effect	NA	No effect/NA	NA	High	No correlation between UIC and TSH or fT4, and no difference in TSH or fT4 between UIC group <50, 50–99, and >100 μg/L.
Borissova et al., 2020 [65]; Bulgaria	Cross-sectional; first, second and third trimester: *n* = 537	170 (95% CI: 161–177)	No effect	NA	NA	NA	High	No difference in TSH between UIC group <150 and 150–249 μg/L.
Chen et al., 2023 [66]; China;	Cross-sectional; first, second, and third trimesters: *n* = 812	148 (104–218)	No effect	No effect/NA	↑/NA	No effect	Moderate	Higher fT4 in UIC group 100–149 μg/L vs. 150–249 μg/L, but no difference in TSH, fT3, or prevalence of thyroid dysfunctions.
Chen et al., 2022 [67]; China	Cross-sectional; pregnant women; first, second and third trimester) recruited from 2 areas: Iodine-sufficient region (Tianjin), *n* = 833 Mildly iodine-deficient region (Wuqiang), *n* = 628	Iodine-sufficient region: 174 (113–249) Mildly iodine-deficient region: 111 (63–167)	No effect	No effect/NA	↓/NA	No effect	High	In the iodine-sufficient region, lower fT4 in UIC group 100–149 vs. 150–249 μg/L. No differences in fT3 or TSH, and no differences in any of the outcomes in the mildly iodine-deficient region.
Corcino et al., 2019 [68]; Brazil	Longitudinal study with repeated cross-sectional data; first trimester: *n* = 243; third trimester: *n* = 100	First trimester: 221 (150–313) Third trimester: 208 (143–327)	No effect	NA	No effect/NA	No effect	High	No difference in TSH or fT4 between UIC group <150 and ≥150 μg/L.
Delshad et al., 2016 [69]; Iran	Cross-sectional; first, second, and third trimesters: *n* = 1,072	87 (47–139)	No effect	NA	No effect/NA	NA	Moderate	No difference in TSH, fT4, or prevalence of thyroid dysfunction between UIC group <100, 100–149, and 150–249 μg/L.
Filipowicz et al., 2023 [70]; Poland	Cross-sectional; *n* = 91	106 (69–156)	↑	No effect/no effect	No effect/no effect	No effect	High	Higher TSH in UIC group <150 vs. 150–249 μg/L.
Fister et al., 2011 [71]; Slovenia	Longitudinal study with repeated cross-sectional data; third trimester and 4 mo postpartum: *n* = 116	UIC:Cr (μg/g): third trimester: 171 4 mo postpartum: 144	No effect	No effect/NA	No effect/NA	NA	High	No correlation between UIC and TSH, fT3, or fT4.
Fu et al., 2017 [72]; China	Cross-sectional; first trimester: *n* = 1764	200 (90–235)	No effect	No effect/NA	No effect/NA	NA	Moderate	No difference in TSH, fT3, or fT4 in UIC group <150 and 150–249 μg/L.
Guo et al., 2020 [73]; China	Cross-sectional; first, second. and third trimesters: *n* = 2378	168 (111–263)	No effect	No effect/NA	No effect/NA	NA	Moderate	No difference in TSH, fT3, or FT4 between UIC groups <100, 100–149, and 150–249 μg/L.
Gustin et al., 2022 [74]; Sweden	Cross-sectional; third trimester: *n* = 531	112 (80–156)	No effect	No effect/no effect	No effect/no effect	NA	Low	No clear associations between UIC and TSH, fT3, fT4, T3, or T4 using regression analyses and correlation.
Habimana et al., 2014 [75]; Congo	Cross-sectional; first, second, and third trimesters: *n* = 225	138 (57–321)	No effect	No effect/NA	No effect/NA	NA	High	No differences in TSH, fT3, or fT4 between UIC group <150 and 150–249 μg/L.
Jiang et al., 2023 [76]; China	Longitudinal study with repeated cross-sectional data; first trimester: *n* = 1332; second trimester: *n* = 1182; third trimester: *n* = 1026	First trimester: 116 (76–172) Second trimester: 111 (74–164) Third trimester: 106 (69–158)	No effect	NA	↓/NA	NA	Low	A positive correlation between UIC and fT4, but the effect size was weak. No difference in TSH between UIC group <150 and ≥150 μg/L.
Knight et al., 2018 [77]; United Kingdom	Cross-sectional; third trimester: *n* = 308	88 (55–157)	No effect	NA	No effect/NA	NA	Moderate	No clear associations between UIC and TSH or fT4 in regression analyses.
Kose Aktas et al., 2022 [78]; Turkey	Longitudinal study with repeated cross-sectional data; first trimester: *n* = 265; second trimester: *n* = 264; third trimester: *n* = 264	First trimester: 96 (range: 1–435) Second trimester: 78 (range: 1–1305) Third trimester: 60 (range: 1–501)	No effect	↓/NA	No effect/NA	NA	High	In first trimester, fT3 was lower in UIC group 50–150 vs. ≥150 μg/L, but not in second and third trimester. No differences were seen in TSH and fT4 between these UIC groups.
Levie et al., 2019 [14]; Sweden	Cross-sectional; first trimester: *n* = 2009	90 (95% range: 38–439)	↓	No effect/no effect	No effect/↑	NA	Low	Lower UIC was associated with a slightly lower TSH and higher T4 in linear regression analyses.
Li et al., 2023 [79]; China	Longitudinal study with repeated cross-sectional data; first trimester: *n* = 1540; second trimester: *n* = 1487; third trimester: *n* = 1306	First trimester: 113 (74–172) Second trimester: 114 (74–168) Third trimester: 105 (68–157)	↑	No effect/NA	No effect/NA	No effect	Low	UIC was not associated with TSH, fT3, or fT4 in the first and second trimesters, and UIC only showed a weak correlation with TSH in the third trimester.
Liu et al., 2022 [80]; China	Cross-sectional; first, second, and third trimesters: *n* = 300	203	↓	No effect/NA	No effect/NA	↑	High	TSH was lower in UIC group <150 vs. 150–249 μg/L, whereas the incidence rate of thyroid disease was higher. No correlation between UIC with fT3 or fT4.
Meng et al., 2013 [36]; China	Cross-sectional; *n* = 326	206	↑	NA	NA	NA	High	Higher TSH in UIC group <100 μg/L vs. 100–199 and 200–299 μg/L.
Moreno-Reyes et al., 2021 [81]; Belgium	Cross-sectional; first and third trimester: *n* = 1229	124 (73–212)	No effect	No effect/NA	No effect/no effect	NA	Moderate	UIC was not associated with fT4, T4, fT3, or TSH in multiple linear regression.
Nazarpour et al., 2020 [83]; Iran	Cross-sectional; first trimester: *n* = 1286	142	No effect	NA	NA/no effect	NA	Moderate	No differences in T4 and TSH between UIC groups <100, 100–150, and 150–250 μg/L.
Næss et al., 2021 [82]; Norway	Longitudinal study with repeated cross-sectional data; second trimester: *n* = 134; third trimester: *n* = 119; 3 mo postpartum: *n* = 111; 6 mo postpartum: *n* = 103	Second trimester: 94 (63–130) Third trimester: 85 (57–123) 3 mo postpartum: 74 (42–130) 6 mo postpartum: 84 (49–120)	No effect	↑/NA	↑/NA	No effect	Low	Lower UIC:Cr, but not UIC, was associated with higher fT3 and fT4 in repeated linear regression models. No associations were seen between TSH and UIC:Cr or UIC.
Pan et al., 2019 [84]; China	Cross-sectional; first, second and third trimester: *n* = 1099	156 (106–225)	No effect	No effect/NA	No effect/NA	NA	High	No differences in TSH, fT3, or fT4 between UIC group <150 and 159–249 μg/L.
Rebagliato et al., 2010 [17]; Spain	Cross-sectional; first and second trimester: *n* = 1844	137 (P2.5: 31; P97.5: >400)	No effect	NA	No effect/NA	NA	Moderate	No association between UIC and TSH or fT4 in multiple linear regression.
Schiller et al., 2020 [18]; Israel	Cross-sectional; first trimester: *n* = 100	49 (16–92)	No effect	No effect/NA	No effect/NA	NA	Moderate	No difference in TSH, fT4, or fT3 between UIC groups <100, 100–150, and >150 μg/L.
Shi et al., 2015 [85]; China	Cross-sectional; first trimester: *n* = 7190	153	No effect	NA	No effect/NA	↑	Low	No difference in TSH or fT4 in UIC groups <100 and 100–149 μg/L vs. 150–249 μg/L, though there was a higher prevalence of overt hypothyroidism in UIC group <100 μg/L.
Silva de Morais et al., 2020 [86]; Brazil	Longitudinal study with repeated cross-sectional data; first and third trimester: *n* = 214	220	No effect	NA	No effect/NA	NA	Moderate	No differences in TSH or fT4 between UIC group <150 and 150–249 μg/L.
Tian et al., 2021 [87]; China	Cross-sectional; first, second, and third trimesters: *n* = 354	119 (85–168)	No effect	No effect/NA	No effect/NA	NA	High	No differences in TSH, fT3, or fT4 between UIC group <150 and 150–249 μg/L.
Wang et al., 2022 [88]; China	Cohort; first trimester: *n* = 1264 (cross-sectional data) Follow-up measurements of thyroid dysfunction in second and third trimesters: *n* = 250	136	No effect	↑/NA	↓/NA	No effect	Moderate	Lower fT4 in UIC group 100–149 μg/L, and higher fT3 in UIC group <100 μg/L vs. UIC group 150–249 μg/L. No differences in TSH between the UIC groups.
Wu et al., 2023 [89]; China	Cross-sectional; first, second, and third trimester: *n* = 744	150 (88–268)	NA	NA	NA	No effect	Low	No differences in thyroid dysfunction between UIC group <150 and 150–250 μg/L.
Wu et al., 2023 [90]; China	Cross-sectional; second trimester: *n* = 562	158 (90–246)	↑	No effect/NA	No effect/NA	↑	High	No differences in TSH, fT3, or fT4 in the UIC group <150 vs. 150–249 μg/L, but the prevalence of isolated hypothyroxinemia was higher. In UIC group <150 μg/L, there was a negative correlation between UIC and TSH.
Xiao et al., 2018 [91]; China	Cross-sectional; first trimester: *n* = 1569	160	No effect	NA	↓/NA	NA	High	Lower fT4 in UIC group <100 vs. 150–249 μg/L. No difference in TSH between the UIC groups.
Yang et al., 2018 [92]; China	Cross-sectional; first, second, and third trimesters: *n* = 1082	204	NA	NA	NA	No effect	High	No differences in prevalence of thyroid dysfunction between UIC groups 50–99, 100–149, and 150–249 μg/L.
Yang et al., 2020 [93]; China	Cross-sectional; first, second, and third trimesters: *n* = 1665	146	No effect	NA	No effect/NA	No effect	High	No differences in TSH, fT4, or thyroid dysfunction rate between UIC group <150 and 150–249 μg/L.
Zha et al., 2023 [94]; China	Cross-sectional; second trimester: *n* = 212	200	No effect	No effect/NA	No effect/NA	NA	High	No differences in fT4, fT3, or TSH between UIC group <150 μg/L and 150–249 μg/L.
Zhang et al., 2019 [95]; China	Longitudinal study with repeated cross-sectional data; GW 6, 12, 24, 32: *n* = 79 at all time-points	107 (84–150)	No effect	No effect/NA	No effect/NA	NA	High	No differences in fT3, fT4, or TSH between UIC groups <100, 100–109, and 110–149 vs. 150–249 μg/L.
Zhang et al., 2022 [96]; China	Cross-sectional; first trimester: *n* = 596	Not given median UIC in total population, but separated in UIC groups: UIC <150 μg/L (*n* = 390): 86 (50–121) UIC 150–249 μg/L (*n* = 206): 187 (167–208)	No effect	NA	No effect/NA	No effect	High	No differences in TSH, fT4, or prevalence of hypothyroxinemia between UIC group <150 and 150–249 μg/L.

Cr, creatinine; fT3, free triiodothyronine; fT4, free thyroxine; GW, gestational week; NA, not applicable; T3, triiodothyronine; T4, thyroxine; TSH, thyroid-stimulating hormone; UIC, urinary iodine concentration.

1 Median (IQR) UIC in the whole study population. If median and/or IQR value is not given, this is specified.

2 The association between iodine status and thyroid function outcomes. The arrows indicate the association between iodine status and thyroid function outcomes, where ↑ indicates higher concentration/prevalence in the iodine-deficient group than that in the adequate-status group, and ↓ indicates lower concentration/prevalence in the iodine-deficient group than that in the adequate-status group.

3 Risk of bias assessed with the Newcastle–Ottawa Scale and scored in the categories high, medium, and low risk of bias. Full risk of bias assessment is given in Supplemental Table 5.

The study populations were from China (*n* = 35), Norway (*n* = 4), Spain (*n* = 3), the United States (*n* = 3), Iran (*n* = 3), and various other countries (*n* = 24 different countries). The total sample size in all studies ranged from 47 to 78,470 participants (*n* = 47–1802 in children and adolescents, *n* = 128–5057 in women of reproductive age, *n* = 412–78,470 in general adults, and *n* = 79–78,470 in pregnant women). All included studies (*N* = 72) used UIC or UIC:Cr as the exposure, and no studies used dietary iodine intake as an exposure. For the outcome, 61 studies included TSH, 47 studies included (f)T3, 52 studies included (f)T4, and 25 studies included data on thyroid dysfunction. Detailed information about the included studies is given in Supplemental Table 4 (including aim of the study, study population, exposure and outcomes assessment, and effect sizes of the results).

The complete results of risk of bias assessment are found in Supplemental Table 5. Using the Newcastle–Ottawa Scale, 39 of the 72 included studies (54%) were judged to have high risk of bias, 19 studies (26%) moderate risk of bias, and 14 studies (19%) low risk of bias (TABLE 2, TABLE 3, TABLE 4, TABLE 5).

### Infants, children, and adolescents

A summary of the findings in infants, children, and adolescents is shown in Table 2. Detailed information about the included studies is given in Supplemental Table 4. Seven studies reported the association between iodine status and thyroid function in infants, young children, and/or adolescents, of which 6 were cross-sectional and 1 had a repeated cross-sectional design. Of these, 1 study included infants [32], 1 study included young children and adolescents <18 y [34], and 5 studies included school-aged children [30,31,33,35,36]. Only 1 of the 7 studies presented measures of thyroid function specifically for individuals with mild-to-moderate iodine deficiency [31]. The rest of the studies did not separate between the degree of iodine deficiency (e.g., provided solely information about thyroid function in a group with UIC < 100 μg/L, corresponding to insufficient iodine status in these population groups, and not specifically mild-to-moderate iodine deficiency). Further, 2 studies did not compare results from groups according to UIC, but rather reported associations between UIC and thyroid function using linear mixed models or correlation coefficients [32,34]. Thus, there were insufficient studies to conduct a meta-analysis of the association between UIC and thyroid function among children and adolescents with mild-to moderate iodine deficiency.

All 7 studies provided information about TSH concentrations. The results showed no clear associations between different levels of UIC and TSH [[30], [31], [32], [33], [34], [35]], except for 1 study, in which TSH concentrations decreased and subsequently increased with the elevation of UIC in school-aged children [36].

Four studies provided information about fT3 or T3 concentrations [[32], [33], [34], [35]]. Of these, 1 found decreased fT3 concentrations in school-aged children with low UIC (either <50 or <100 μg/L) compared with that in children with UIC above these cutoff values, although the fT3 concentrations for all children fell within normal reference ranges [33]. Three of the studies found no or negligible associations between different levels of UIC and fT3/T3 [32,34,35].

Four of the studies included information about fT4 or T4 concentrations, but the results did not indicate a clear association between fT4/T4 concentrations and UIC [[32], [33], [34], [35]]. None of the studies in children and adolescents reported on the prevalence of thyroid dysfunction.

### Women of reproductive age (including lactating women)

A summary of the findings for women of reproductive age (including lactating women) is shown in Table 3. Detailed information about the included studies is given in Supplemental Table 4. Five studies examined possible associations between iodine status and thyroid function in women of reproductive age (range 15–45 y) [[36], [37], [38], [39], [40]], of which 2 studies included lactating women [36,39]. All 5 studies had a cross-sectional design.

All 5 studies provided information about TSH concentrations. One study found a positive association between TSH concentrations and UIC/Cr, but not with UIC [40]. In the other 4 studies, results showed no clear difference in TSH concentrations between the iodine-deficient group (<100 μg/L) and the iodine-adequate group (100–199 or 100–299 μg/L) [[36], [37], [38], [39]].

Four studies provided information about fT4 or T4 concentrations. Of these, 1 study found lower fT4 concentrations in the iodine-deficient group (<100 μg/L) than those in the iodine-adequate group (100–299 μg/L) [38], whereas the other 3 studies found no clear association between UIC and fT4/T4 [37,39,40].

One study reported on thyroid dysfunction and found no relationship between the prevalence of hyperthyroidism or hypothyroidism and iodine status [38]. None of the studies in women of reproductive age reported measurements of fT3 or T3 concentrations.

### General adults

A summary of the findings in general adults is shown in Table 4. Detailed information about the included studies is given in Supplemental Table 4. Twenty studies examined the association between iodine status and thyroid function in general adults [36,[41], [42], [43], [44], [45], [46], [47], [48], [49], [50], [51], [52], [53], [54], [55], [56], [57], [58], [59]], of which 1 study included only older adults [41] and 2 studies included both children and adults (results reported together) [48,49]. All studies had a cross-sectional design.

In total, 12 of the 20 studies included measurements of TSH concentrations [36,[40], [41], [42], [43],^47^,^48^,[52], [53], [54], [55],^58^,^59^]. Five of these did not show an association between different levels of UIC and TSH concentrations [43,[53], [54], [55],^58^], whereas results from 4 studies reported higher TSH concentrations in the iodine-deficient group (UIC < 100 μg/L) than those in the iodine-adequate group (>100, 100–199, or 100–299 μg/L) [36,42,47,52]. In 3 studies, TSH was lower in groups classified as iodine-deficient group than those with adequate iodine status [41,48,59].

Seven of the studies examined fT3 or T3 concentrations [[41], [42], [43], [47], [52], [53], [55]], of which 1 study [47] found higher T3 concentrations in the iodine-deficient group (<100 μg/L) compared to the iodine-adequate group (>100 μg/L). The rest of the studies found no clear association between fT3/T3 concentrations and UIC.

Further, 8 studies examined fT4 or T4 concentrations [[41], [42], [43],^47^,^48^,^52^,^53^,^55^]. Of these, 2 studies found higher fT4/T4 concentrations in the iodine-deficient group (<100 μg/L) than those in the iodine-adequate group (100–199 or >100 μg/L) [42,47], whereas 1 study found lower fT4 concentrations in men, but not in women, in an area with mild iodine deficiency (median UIC: 55 μg/L) than those in an area with adequate status (median UIC: 160 μg/L) [41]. Another study found a negative association between fT4 concentrations and UIC:Cr ratio, but not with UIC [53]. The rest of the studies found no clear difference in fT4/T4 concentrations with different levels of UIC [43,48,52,53,55]. Ten studies reported on the prevalence of thyroid dysfunction [41,[44], [45], [46],[49], [50], [51],^56^,^57^,^59^]. Of these, 6 studies found a higher prevalence of thyroid dysfunction in the group with mild-to-moderate or insufficient iodine status than that in the group with adequate iodine status [41,[45], [46],^50^,^56^], whereas 4 studies found no differences in the prevalence of thyroid dysfunction between the 2 groups [49,51,57,59].

### Pregnant women

A summary of the findings in pregnant women is shown in Table 5. Detailed information about the included studies is given in Supplemental Table 4. A total of 43 included studies examined possible associations between iodine status and thyroid function in pregnant women [[14], [15], [16], [17], [18],^34^,[60], [61], [62], [63], [64], [65], [66], [67], [68], [69], [70], [71], [72], [73], [74], [75], [76], [77], [78], [79], [80], [81], [82], [83], [84], [85], [86], [87], [88], [89], [90], [91], [92], [93], [94], [95], [96]]. Of these, 30 had a cross-sectional design [14,15,17,18,36,61,[64], [65], [66], [67],^69^,^70^,[72], [73], [74], [75],^77^,^80^,^81^,[83], [84], [85],^87^,[89], [90], [91], [92], [93], [94],^96^], 12 had a repeated cross-sectional design [16,60,62,63,68,71,76,78,79,82,86,95], and 1 was a cohort study [88].

A total of 40 studies among pregnant women included measurements of TSH concentrations. Of these, 33 studies found no clear association between different levels of UIC and TSH concentrations [15,17,18,[61], [62], [63],[64], [65], [66], [67], [68], [69],[71], [72], [73], [74], [75], [76], [77], [78],[81], [82], [83], [84], [85], [86], [87], [88],^91^,[93], [94], [95], [96]]. Further, 4 studies found higher TSH concentrations in the iodine-deficient group (UIC: <100 or <150 μg/L) than those in the iodine- adequate group (UIC: 150–249 or 200–299 μg/L) [36,70,79,90], whereas 3 studies found lower TSH concentrations in the iodine-deficient group (UIC <150 μg/L) than those in the iodine-adequate group (UIC: 150–249 μg/L) [14,60,80].

A total of 25 studies reported fT3 or T3 concentrations in pregnant women [[14], [15], [16],^18^,^60^,^62^,^66^,^67^,[70], [71], [72], [73], [74], [75],[78], [79], [80], [81], [82],^84^,^87^,^88^,^90^,^94^,^95^]. Of these, 19 studies found no clear association between UIC and fT3/T3 concentrations [14,18,62,[66], [67], [70], [71], [72], [73], [74], [75],[79], [80], [81],[84], [87],[90], [94], [95]]. Further, 5 studies found higher fT3/T3 concentrations in the iodine-deficient group (UIC: 100–149 or <150 μg/L) than those in the iodine-adequate group (UIC: >150 or 150–249 μg/L) [15,16,60,82,88], whereas 1 study found lower fT3 concentrations in the iodine-deficient group (UIC 50–150 μg/L) than those in the group with adequate status (UIC ≥150 μg/L) [78].

A total of 38 studies measured fT4 or T4 concentrations in pregnant women [[14], [15], [16], [17], [18],[60], [61], [62],^64^,[66], [67], [68], [69], [70], [71], [72], [73], [74], [75], [76], [77], [78], [79], [80], [81], [82], [83], [84], [85], [86], [87], [88],^90^,^91^,[93], [94], [95], [96]]. Of these, 29 studies found no clear association between UIC and fT4/T4 concentrations [17,18,[60], [61], [62],^64^,[68], [69], [70], [71], [72], [73], [74], [75],[77], [78], [79], [80], [81],[83], [84], [85], [86], [87],^90^,[93], [94], [95], [96]]. Furthermore, 5 studies found higher fT4 or T4 concentrations in the iodine-deficient group (UIC: 100–149 or <150 μg/L) than those in the iodine-adequate group (UIC: >150 or 150–249 μg/L) [[14], [15], [16],^66^,^82^], whereas 4 studies found lower fT4 concentrations [67,76,88,91] when comparing the iodine-deficient group (UIC: 100–149 μg/L or <100 μg/L) with the iodine-adequate group (UIC: 150–249 μg/L or ≥150 μg/L).

A total of 14 studies reported the prevalence of thyroid dysfunction in pregnant women [[66], [67], [68],^70^,^79^,^80^,^82^,^85^,[88], [89], [90],^92^,^93^,^96^]. Of these, 11 studies found no difference in the prevalence of thyroid dysfunction [[66], [67], [68],^70^,^79^,^82^,^88^,^89^,^92^,^93^,^96^] in the iodine-deficient group compared with that in the iodine-adequate group. However, 1 study found a higher prevalence of any thyroid disease [80], 1 study found a higher prevalence of overt hypothyroidism [85], and 1 study found a higher prevalence of isolated hypothyroxinaemia [90] in the iodine-deficient group (UIC: <100 or <150 μg/L) compared with those in the iodine-adequate group (UIC: 150–249 μg/L).

### Meta-analysis

**FIGURE 2:**
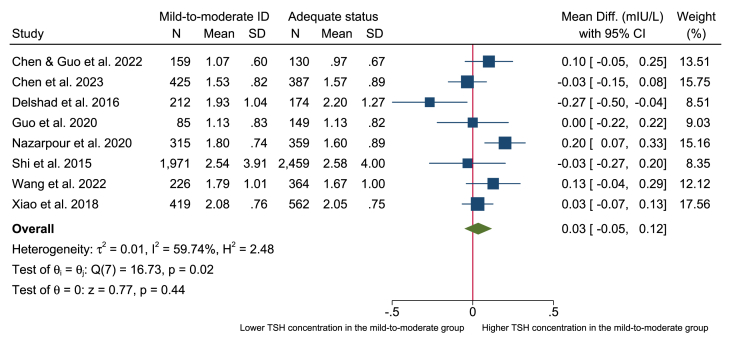
Forest plot of the mean difference in TSH (mIU/L) in pregnant women with mild-to-moderate iodine deficiency (defined as UIC = 50–150 μg/L) compared with adequate iodine status (defined as UIC = 150–249 μg/L). Data were analyzed using random-effect meta-analysis with the restricted maximum likelihood technique. ID, iodine deficiency; TSH, thyroid-stimulating hormone; UIC, urinary iodine concentration.

**FIGURE 3:**
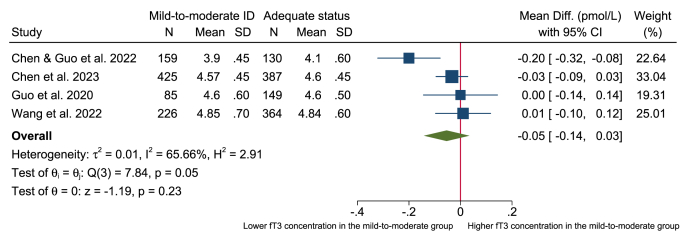
Forest plot of the mean difference in fT3 (pmol/L) in pregnant women with mild-to-moderate iodine deficiency (defined as UIC = 50–150 μg/L) compared with adequate iodine status (defined as UIC = 150–249 μg/L). Data were analyzed using random-effects meta-analysis with the restricted maximum likelihood technique. ID, iodine deficiency; fT3, free triiodothyronine; UIC, urinary iodine concentration.

**FIGURE 4:**
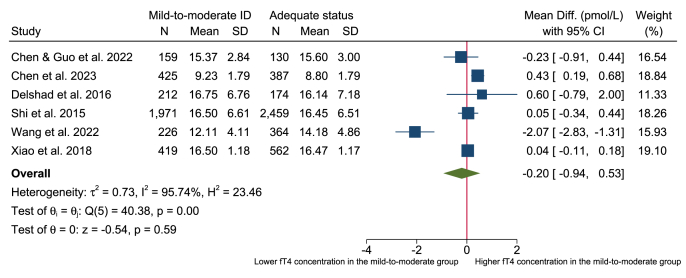
Forest plot of the mean difference in fT4 (pmol/L) in pregnant women with mild-to-moderate iodine deficiency (defined as UIC = 50–150 μg/L) compared with adequate iodine status (defined as UIC = 150–249 μg/L). Data were analyzed using random-effects meta-analysis with the restricted maximum likelihood technique. ID, iodine deficiency; fT4, free thyroxine; UIC, urinary iodine concentration.

Eight studies among pregnant women measured thyroid function data specifically for groups with mild-to-moderate iodine deficiency compared with those with adequate status and were included in meta-analysis—8 for TSH [66,67,69,73,83,85,88,91], 4 for fT3 [66,67,73,88], and 6 for fT4 [66,67,69,85,88,91]. All included studies had a cross-sectional design. The meta-analyses showed no significant difference in the concentration of TSH, fT3, or fT4 in pregnant women with mild-to-moderate iodine deficiency (UIC: 50–149 μg/L) compared with those in pregnant women with adequate status (UIC: 150–249 μg/L) [mean difference (95% CI): TSH, 0.03 mIU/L (−0.05, 0.12 mIU/L); fT3, 0.05 pmol/L (−0.14, 0.03 pmol/L); fT4, −0.20 pmol/L (−0.94, 0.53 pmol/L)] (FIGURE 2, FIGURE 3, FIGURE 4, respectively). The proportion of heterogeneity between the included studies were moderate (TSH: *I*^2^ = 59.7%; fT3: *I*^2^ = 65.7%) or high (fT4: *I*^2^ = 95.7%).

For the other population groups (infants, children and adolescents, women of reproductive age, and adults), there were not sufficient data for meta-analysis comparing thyroid function data in population groups with mild-to-moderate iodine deficiency with groups with adequate status. Publication bias was assessed by visual inspection of funnel plots and given in Supplemental Figures 1–3.

### Grading of evidence

Using the WCRF criteria for grading the evidence, we graded the overall evidence of an association between mild-to-moderate iodine deficiency and thyroid function as limited—no conclusion, for all thyroid function outcomes and all population groups. This conclusion was based on the following: *1*) except for 1 study, all studies had a cross-sectional design; *2*) most of the included studies reported no clear association between thyroid function outcomes and iodine status; and *3*) the included studies that found an association between iodine status and thyroid function were inconsistent in the direction of effects.

## Discussion

This systematic review and meta-analysis assessed the evidence of the association between mild-to-moderate iodine deficiency and thyroid function in different population groups. We included 72 observational studies, and no clear association between mild-to-moderate iodine deficiency and thyroid function was apparent in the population groups of children and adolescents, women of reproductive age, general adults, or in pregnant women. The evidence was limited—no conclusion, where most of the studies reported no clear association, and the few studies that indicated an association were inconsistent in the direction of effects. In addition, almost all included studies had a cross-sectional design. Furthermore, the evidence base for comparing thyroid function within study populations with mild-to-moderate iodine deficiency and adequate iodine status was limited as few studies presented results for both categories. Consequently, only 8 studies were eligible for inclusion in the meta-analyses, and these were limited to pregnant women. The meta-analyses revealed no significant differences in thyroid function outcomes (TSH, fT3, and fT4) (FIGURE 2, FIGURE 3, FIGURE 4) between pregnant women with mild-to-moderate iodine deficiency and those with adequate iodine status.

To our knowledge, we are not aware of other systematic reviews published in the scientific literature, which have explored the association between mild-to-moderate iodine deficiency and thyroid function in the population groups of children and adolescents, women of reproductive age, and general adults. However, in a report from the Norwegian Scientific Committee on Food and Environment (VKM) from 2020, which assessed risks and benefits of salt iodization, systematic literature reviews were performed on the potential effects of mild-to-moderate iodine deficiency on thyroid function outcomes across different life stages [97]. Similar to our findings, the VKM report concluded that there was “limited—no conclusion” evidence to support that mild-to-moderate iodine deficiency was associated with thyroid dysfunction in school-aged children, nonpregnant adults, and pregnant women.

In pregnant women, previously published systematic reviews have examined possible effects of iodine supplementation on thyroid function outcomes, of which all have reported inconsistent results [3,6,7,[20], [21], [22]]. The systematic review by Dineva et al. [6], which specifically focused on possible effects of iodine supplementation in mildly to moderately iodine-deficient pregnant women, concluded that most studies showed no effect of maternal iodine supplementation on maternal or infant TSH or fT4 concentrations. However, in a meta-analysis restricted to 2 of the included RCTs, iodine supplementation significantly reduced maternal TSH concentrations [6]. To date, the largest RCT on iodine supplementation in mildly to moderately iodine deficient pregnant women, which was also included in the meta-analysis by Dineva et al. [6], showed no difference in mean maternal TSH and fT4 concentrations between the supplement and control groups [98]. Although a slightly lower mean maternal T3 concentration was reported in the supplement group 6 wk postpartum, the authors concluded that iodine supplementation did not improve maternal or newborn thyroid function [98]. The findings from the above-mentioned systematic reviews and RCTs on mild-to-moderate iodine deficiency and thyroid function are in line with the results presented in this review.

Several factors may explain why we did not find a clear association between mild-to-moderate iodine deficiency and thyroid function in this work. A key issue is the uncertainty in the exposure, specifically UIC, reflecting recent iodine status. As no individual marker for iodine status exists, most of the included studies used the median UIC from spot urine samples, which is recommended for assessing iodine status in population groups [23]. However, using the median UIC from spot samples as an indicator of iodine nutrition can be challenging as it may be affected by random error and does not represent usual intake, which is what we want to capture [99]. Therefore, making UIC groups based on the individual UIC measured by a spot urine sample increases risk of misclassifying individuals. Consequently, defining a population as iodine deficient based on UIC cutoffs does not mean that the entire population is iodine deficient. However, compared with a population defined as iodine replete, the proportion with clinical consequences related to thyroid function will likely be larger in the population defined as iodine deficient. Our analytical strategy may not capture such tail effects, and this can mask the possible associations between iodine status and thyroid function outcomes, which may lead to regression dilution bias shifting the effect measures toward the zero [100]. One way to reduce risk misclassification bias is to examine the exposure variable on a continuous scale [101]. In this systematic review, we also examined the included studies that analyzed UIC or UIC:Cr on a continuous scale (*n* = 16 studies). Of these, 8 studies found an association with thyroid function outcomes [14,15,34,53,60,79,82,90], whereas 8 did not [32,62,63,71,74,77,81,94], thus showing heterogeneous results when not grouping the exposure variable into categories.

Since the thyroid can store iodine for ∼3 months of hormone production, an association might only be expected in participants with consistently low iodine intake over time [102]. This can be assessed by obtaining repeated measurements from each participant (or a subsample) [100]. In the presented systematic review, most included studies had a cross-sectional design, and only 13 of 72 studies included repeated measurements of UIC. Even in these studies, no clear associations between mild-to-moderate iodine deficiency and thyroid function outcomes were observed [16,32,60,62,63,68,71,76,78,79,82,86,95].

Furthermore, there is uncertainty and debate related to established UIC cutoffs for different population groups [2,103], and no cutoffs for mild-to-moderate iodine deficiency has been set for children <6 y of age, pregnant, or lactating women. The latest guide for assessment of iodine status in populations was published in 2007 [23], and the UIC thresholds are currently under revision by the WHO. The absence of established thresholds for mild-to-moderate iodine deficiency makes it challenging to assess its effects on thyroid function and other health outcomes. Furthermore, the wide-ranging definition of mild-to-moderate iodine deficiency could also affect thyroid function outcomes differently, as it is possible that mild and moderate iodine deficiency affects thyroid function with different degrees of severity. For example, the most used definition of mild-to-moderate iodine deficiency in pregnant women, a median UIC in the range of 50–150 μg/L [24], is rather broad, and one would likely expect more severe consequences in a population with a median UIC of 50 μg/L compared with those in, for instance, a population with a median UIC of 120 μg/L. In our systematic review, most of the included studies were in the category of mild iodine deficiency, and few were in the category of moderate iodine deficiency, which again might have biased our results toward a null effect.

Considering the limitations in UIC, we examined all included studies that assessed iodine intake from dietary assessment methods, to explore whether the association with thyroid function could be different when using other methods for assessing iodine exposure. Of the 3 studies that included both UIC and estimated iodine intake, 1 study [85] found that TSH concentrations were neither associated with UIC nor with estimated iodine intake. Another study [82] found that lower UIC and estimated iodine intake were associated with higher fT4 concentrations, whereas for TSH, a positive association was found only for estimated iodine intake. In the third study [15], TSH concentrations were not associated with either UIC or iodine intake, although fT4 concentrations were inversely associated with UIC, but not with dietary iodine intake [15]. Thus, we did not find any clear indication that estimated iodine intake by dietary assessments methods had a stronger or weaker association with thyroid function than UIC. However, as we included only 3 studies assessing both measures, this should be a topic of further investigation.

Another consideration that may have contributed to masking a possible association between mild-to-moderate iodine deficiency and thyroid function is related to the inconsistency in how thyroid function outcomes and thyroid dysfunction were analyzed and reported across studies. The reference ranges and definitions used in the included studies varied considerably, a matter of ongoing debate [104,105]. Furthermore, the studies reported thyroid function outcomes using different units. To enable meta-analyses, statistical converters derived mean differences for comparing thyroid function outcomes in populations with mild-to-moderate iodine deficiency and that in those with adequate status. This approach might have introduced errors. For instance, the distribution of TSH is often skewed, meaning that the conversion from medians and percentiles to means and SDs may not fully capture the underlying distribution. Future research would benefit from standardized reporting practices that include both mean and SD alongside medians and percentiles, to facilitate comparisons between studies. Despite the above-mentioned challenges in investigating the potential impact of mild-to-moderate iodine deficiency on thyroid function, it is also possible that in a state of mild-to-moderate iodine deficiency, changes in thyroid homeostasis can compensate to ensure euthyroidism and keep levels of TSH, fT3, and fT4 within the normal range [106].

Although the effect of mild-to-moderate iodine deficiency on thyroid outcomes remain uncertain, the evidence on child developmental outcomes were found to be limited—suggestive in the assessment by the VKM [97]. This conclusion was based on evidence from several observational studies, which generally found a consistent direction of association [97]. However, as previously mentioned, the results from RCTs evaluating possible effects of iodine supplementation are inconsistent. In the aforementioned RCT with iodine supplementation in mildly to moderately iodine-deficient pregnant women, no effect was found on child developmental outcomes [98]. Conversely, 2 RCTs found that iodine supplementation in mildly and moderately iodine-deficient school-age children improved cognition [107,108]. In the study of moderately iodine-deficient children from Albania, Zimmermann et al. [108] explained the improved cognition scores with an increase in total T4 concentrations in the intervention group. However, in another study of mildly iodine-deficient children from New Zealand, Gordon et al. [107] were not able to demonstrate that the improvement in cognition was mediated via changes in total T4. Consequently, a challenge with the current evidence on developmental outcomes is the lack of plausibility in the biological mechanisms of how mild-to-moderate iodine deficiency may cause impaired development [109]. It is possible that the adverse effects of iodine deficiency may appear along a continuum of decreasing long-term iodine intake, making the association between mild-to-moderate iodine deficiency and thyroid function and further child development difficult to prove with the methodological weaknesses of the current iodine biomarker. However, the possibility that mild-to-moderate iodine deficiency does not cause impaired child development should also be considered.

### Strengths and limitations

In this review, we followed the established rigorous guidelines for completion of a systematic review. The strengths include a preregistered detailed study protocol, a comprehensive literature search across 3 databases, as well as duplicate study selection, risk of bias assessments, and data extraction completed independently by 2 authors. As a result, we likely identified the most relevant literature in this area of research.

There are also some limitations that should be mentioned, in addition to the aforementioned methodological issues with UIC and thyroid function markers. This systematic review and meta-analyses included only observational studies, and except for 1 study, all were of cross-sectional design. Furthermore, a limited number of studies were included in the meta-analyses owing to heterogeneity between the included studies. Thus, it was only possible to perform meta-analyses in the population group of pregnant women, with only 8 studies included. Another limitation is that we did not conduct hand-searching for potential additional literature or contact the authors of the included articles to request additional data. Owing to the limited number of studies included in the meta-analyses, we were also not able to perform subgroup analyses or test for publication bias. Furthermore, the quality assessment of the included studies suggested that the majority of the included articles were of poor quality (more than half of the studies were graded as high risk of bias), constituting a limitation in the overall evidence. Additionally, few of the included studies were addressed to study the effect of the association between mild-to-moderate iodine deficiency with thyroid function, and several studies included this as secondary outcomes. Thus, the study designs and measurements were not developed solely to answer the main objective of this systematic review.

Overall, the evidence of the association between mild-to-moderate iodine deficiency and thyroid function outcomes was graded as limited—no conclusion, meaning that no firm conclusion can be made. Although the evidence was limited, our findings are consistent with previous systematic reviews and RCTs. However, this does not rule out the possibility of an association between mild-to-moderate iodine deficiency and thyroid function. Therefore, further investigation with high-quality studies is needed to better understand the potential effects of mild-to-moderate iodine deficiency. Nevertheless, it is also important to acknowledge that the current evidence suggests that thyroid hormones are in general adequately maintained if mild-to-moderate iodine deficiency is present and that, currently, no clear association between them are seen.

## Conclusion

In this systematic review, we were unable to demonstrate any association between mild-to-moderate iodine deficiency and thyroid function. Given the high global prevalence of mild-to-moderate iodine deficiency, particularly in pregnant women and women of childbearing age, understanding its impact on thyroid function—and other health outcomes—is of significant public health importance. Iodine is critical for thyroid function, and thyroid hormones are likely the best biomarkers reflecting health outcomes of iodine deficiency. Exploring the association between mild-to-moderate iodine deficiency and thyroid function is important as it may impact iodine supplementation and fortification programs for populations with mild-to-moderate iodine deficiency.

## Author contributions

The author’s responsibilities were as follows – SNS, IA, TAS, MWM: had the research idea; TEA, SNS: developed the study protocol with input from all authors; TEA, SNS: performed the literature search, screened the abstracts, extracted the data from the selected studies, and performed risk of bias assessments; TEA, SNS, IA: wrote the first draft of the paper; TEA: performed the meta-analyses; IA, TAS, KSB: supervised; and all authors: reviewed, edited, and approved the final manuscript.

## Data availability

Data described in the manuscript, code book, and analytic code can be made available upon request.

## Funding

The authors reported no funding received for this study.

## Conflict of interest

The authors report no conflicts of interest.
